# Supplementary material for: Multiple transgressions and slow evolution shape the phylogeographic pattern of the blind cave-dwelling shrimp Typhlocaris
Source: PeerJ. 2018 Jul 23;6:e5268. doi: 10.7717/peerj.5268 (PMC6061184; doi:10.7717/peerj.5268)
Supplement: Supplemental Information 1 [file peerj-06-5268-s001.docx]

| **Locus** | **Primer name** | **Primer sequence (5′ → 3′)** | **PCR Profile** | **Source** |
| --- | --- | --- | --- | --- |
| 12S rRNA | 12SF | GAAACCAGGATTAGATACCC | 40 cycles of 30s at 94°C, 45s at 47°C and 15s at 72°C, followed by a ﬁnal extension of 7 min at 72°C | Kocher et al. (1989) modiﬁed by Mokady et al. (1999) |
|  | 12SR | TTTCCCGCGAGCGACGGGCG |  |  |
| 16S rRNA | 16SAR | CGCCTGTTTATCAAAAACAT | 40 cycles of 25s at 92°C, 90s at 50°C and 25s at 72°C, followed by a ﬁnal extension of 7 min at 72°C | Palumbi (1996) |
|  | 16SBR | CCGGTCTGAACTCAGATCACGT |  |  |
| 18S rRNA | 18SF | TAATGATCCTTCCGCAGGTT | 35 cycles of 70s at 92°C, 90s at 54°C and 50s at 72°C, followed by a ﬁnal extension of 7 min at 72°C | Spears et al. (1994) |
|  | 18SR | CCTGGTTGATCCTGCCAG |  |  |
| 28S rRNA | 28SA | GACCCGTCTTGAAGCACG | 40 cycles of 25s at 92°C, 90s at 50°C and 25s at 72°C, followed by a ﬁnal extension of 7 min at 72°C | Whiting (2002) |
|  | 28SB | TCGGAAGGAACCAGCTAC |  |  |
| Cytochrome Oxidase Subunit 1 (COI) | LCO1490 | GGTCAACAAATCATAAAGATATTGG | initial 2 min denaturation at 95°C, 35 cycles of 90s denaturation, 30s annealing at 50 °C, 45s at 72°C, and a final 10 min extension at 72°C | Folmer et al. (1994) |
|  | HCO2198 | TAAACTTCAGGGTGACCAAAAAATCA |  |  |
| Histone 3 (H3) | H3F | ATGGCTCGTACCAAGCAGACVGC | 40 cycles of 25s at 92°C, 90s at 50°C and 25s at 72°C, followed by a ﬁnal extension of 7 min at 72°C | Colgan et al. (1998) |
|  | H3R | ATATCCTTRGGCATRATRGTGAC |  |  |
| ITS2-28S rRNA | ITS-Typh1f | GGTCGTCTAGAGGAAGTAAAAGTC | 35 cycles of 30s at 94°C, 30s at 54°C and 45s at 72°C, followed by a ﬁnal extension of 7 min at 72°C | Newly designed for this study. |
|  | ITS-Typh1r | TTCCCGAACACCACATTGCACGACG |  |  |

**REFERENCES**

Colgan D, McLauchlan A, Wilson G, Livingston S, Edgecombe G, Macaranas J, Cassis G, and Gray M. 1998. Histone H3 and U2 snRNA DNA sequences and arthropod molecular evolution. *Australian Journal of Zoology* 46:419-437.

Folmer O, Black M, Hoeh W, Lutz R, and Vrijenhoek R. 1994. DNA primers for amplification of mitochondrial cytochrome c oxidase subunit I from diverse metazoan invertebrates. *Molecular marine biology and biotechnology* 3:294-299.

Kocher TD, Thomas WK, Meyer A, Edwards SV, Pääbo S, Villablanca FX, and Wilson AC. 1989. Dynamics of mitochondrial DNA evolution in animals: amplification and sequencing with conserved primers. *Proceedings of the National Academy of Sciences* 86:6196-6200.

Mokady O, Loya Y, Achituv Y, Geffen E, Graur D, Rozenblatt S, and Brickner I. 1999. Speciation versus phenotypic plasticity in coral inhabiting barnacles: Darwin's observations in an ecological context. *Journal of Molecular Evolution* 49:367-375.

Palumbi SR. 1996. Nucleic acids II: the polymerase chain reaction. *Molecular systematics*:205-247.

Spears T, Abele LG, and Applegate MA. 1994. Phylogenetic study of cirripedes and selected relatives (Thecostraca) based on 18S rDNA sequence analysis. *Journal of Crustacean Biology* 14:641-656.

Whiting MF. 2002. Mecoptera is paraphyletic: multiple genes and phylogeny of Mecoptera and Siphonaptera. *Zoologica Scripta* 31:93-104.
