# Supplementary material for: Multiple transgressions and slow evolution shape the phylogeographic pattern of the blind cave-dwelling shrimp Typhlocaris
Source: PeerJ. 2018 Jul 23;6:e5268. doi: 10.7717/peerj.5268 (PMC6061184; doi:10.7717/peerj.5268)
Supplement: Supplemental Information 2 [file peerj-06-5268-s002.docx]

| Gene | ***Typhlocaris ayyaloni*** | ***Typhlocaris galilea*** | ***Typhlocaris salentina*** |
| --- | --- | --- | --- |
| 12S rRNA | KY593415; KY593416 | KY593417; KY593418 | KY593419; KY593420 |
| 16S rRNA | KY593421; KY593422 | KY593423; KY593424 | KY593425; KY593426 |
| 18S rRNA | KY593427 | KY593428 | KY593429 |
| 28S rRNA | KY593430 | KY593431 | KY593432 |
| Cytochrome Oxidase Subunit 1 (COI) | KY593433; KY593434; KY593436 | KY593437; KY593438; KY593439 | KY593440; KY593441; KY593442 |
| Histone 3 (H3) | KY593443; KY593444 | KY593445; KY593446 | KY593447; KY593448 |
| ITS2-28S rRNA | KY593449; KY593450 | KY593451; KY593452 | KY593453; KY593454 |
